# Performance Evaluation of Missing-Value Imputation Clustering Based on a Multivariate Gaussian Mixture Model

**DOI:** 10.1371/journal.pone.0161112

**Published:** 2016-08-23

**Authors:** Jing Xiao, Qiongqiong Xu, Chuanli Wu, Yuexia Gao, Tianqi Hua, Chenwu Xu

**Affiliations:** 1 Department of Epidemiology and Medical Statistics, School of Public Health, Nantong University, Nantong, 226019, China; 2 Jiangsu Key Laboratory of Crop Genetics and Physiology/Co-Innovation Center for Modern Production Technology of Grain Crops, Key Laboratory of Plant Functional Genomics of the Ministry of Education, Yangzhou University, Yangzhou, 225009, China; Southwest University, CHINA

## Abstract

**Background:**

It is challenging to deal with mixture models when missing values occur in clustering datasets.

**Methods and Results:**

We propose a dynamic clustering algorithm based on a multivariate Gaussian mixture model that efficiently imputes missing values to generate a “pseudo-complete” dataset. Parameters from different clusters and missing values are estimated according to the maximum likelihood implemented with an expectation-maximization algorithm, and multivariate individuals are clustered with Bayesian posterior probability. A simulation showed that our proposed method has a fast convergence speed and it accurately estimates missing values. Our proposed algorithm was further validated with Fisher’s Iris dataset, the Yeast Cell-cycle Gene-expression dataset, and the CIFAR-10 images dataset. The results indicate that our algorithm offers highly accurate clustering, comparable to that using a complete dataset without missing values. Furthermore, our algorithm resulted in a lower misjudgment rate than both clustering algorithms with missing data deleted and with missing-value imputation by mean replacement.

**Conclusion:**

We demonstrate that our missing-value imputation clustering algorithm is feasible and superior to both of these other clustering algorithms in certain situations.

## Introduction

Clustering analysis, as a multivariate statistical method, refers to the process of classifying a set of observations into subsets, called clusters, such that observations in the same cluster are similar in certain respects [1–3]. Clustering is widely used in medical sciences, for instance when clustering diseases or gene-expression profiles. Clustering methods usually fall into two categories: hierarchical clustering methods [4], which are used for clustering datasets with small size [5,6]; and dynamic clustering methods, such as K-means [7,8] and self-organizing maps [9], which begin with an initial partitioning of the individuals and iteratively move individuals from one cluster to another until the criterion of convergence is met. With dynamic clustering, the number of clusters must be specified in advance [10]. Dynamic algorithms are mostly heuristically motivated, and they do not require an underlying statistical model. Nevertheless, selection of the “correct” number of clusters and of the best clustering method remains a topic for discussion. Model-based clustering methods [11–13] are a type of dynamic clustering based on the hypothesis that the whole dataset is a finite mixture of the same type of distribution with different sets of parameters, such as a finite mixture of a multivariate Gaussian distribution. Compared with “heuristic” algorithms, one obvious advantage to model-based clustering is that objective statistical criteria—such as the Akaike information criterion (AIC) or the Bayesian information criterion (BIC) [14]—are used to determine the number of clusters.

Missing data present a problem for medical research, given that the data are often supplied retrospectively and from various sources [15]. In particular, missing data are a frequent occurrence in microarray experiments and in medical research. Missing values are especially common in large-scale studies involving dozens of variables and hundreds of individuals. Indeed, many clustering methods require a full set of data. Individuals with missing values are either rejected or been estimated prior to the analysis. Consequently, several missing-value imputation methods have been developed [16–20], such as mean substitution, regression imputation, fuzzy c-means (FCM) clustering of incomplete data [21], and Gaussian mixture model-based missing-value imputation classification [22]. In this study, we propose a dynamic method for a model-based missing-value imputation clustering algorithm. With our proposed method, missing values are estimated iteratively until arithmetic convergence is reached during the process of clustering individuals. We used 12 simulated datasets. Each of these datasets had two versions: a complete version and a version where 10% of the individuals had at least one variable missing. We used these datasets to evaluate the clustering accuracy and the accuracy and precision of cluster-parameter estimators. We compared our proposed algorithm based on the missing-value imputation according to the maximum likelihood estimation (a “pseudo-complete” dataset) with a model-based clustering algorithm using the complete dataset and another two model-based clustering algorithms, one using a dataset with missing values deleted and the other imputing the missing values by mean replacement. In addition, we compared our algorithm with the FCM clustering method using real datasets—viz., Fisher’s Iris dataset, the Yeast Cell-cycle Gene-expression dataset, and the CIFAR-10 images dataset.

## Methods

### Multivariate Gaussian mixture model

The dataset is arranged as an *n* × *k* matrix denoted by Y, where *n* is the number of individuals and *k* is the number of variables. Let *y*_*ij*_ be the observed value of the *i*-th individual in the *j*-th variable, for *i* = 1,2,…, *n* and *j* = 1,2,…, *k*. Let Y_*i*_ = (*y*_*i*1_,*y*_*i*2_,…,*y*_*ik*_)^T^ be the *i*-th column in matrix Y^T^—i.e., a *k* × 1 vector of the data for individual *i* under all variables. The value of *y*_*ij*_ across all the *k* variables represents the expression level of the *i*-th individual. Under a finite multivariate Gaussian mixture model, each Y_*i*_ is assumed to follow a *k*-dimensional Gaussian mixture distribution. Mathematically, the mixture distribution for *c* clusters is as follows:

$$f(Y_{i})=\sum_{l=1}^{c}p_{l}f_{l}(Y_{i}),$$ (1)

where $f_{l}(Y_{i})={\left(2\pi \right)}^{-k/2}\;{\left|S_{l}\right|}^{-1/2}\;\exp\left[-(1/2)(Y_{i}-\mu_{l})S_{l}^{-1}{\left(Y_{i}-\mu_{l}\right)}^{\text{T}}\right]$ is the probability density function for the *l*-th *k*-dimensional Gaussian distribution with mean vector μ_*l*_ = (*μ*_*l*1_, *μ*_*l*2_,…, *μ*_*lk*_) and variance-covariance matrix $S_{l}=\left[\begin{array}{cccc} \sigma_{l1}^{2} & \sigma_{l12} & \cdots & \sigma_{l1k}\\ \sigma_{l21} & \sigma_{l2}^{2} & \cdots & \sigma_{l2k}\\ \vdots & \vdots & \ddots & \vdots \\ \sigma_{lk1} & \sigma_{lk2} & \cdots & \sigma_{lk}^{2} \end{array}\right]$, for *l* = 1,2,…,*c*. Moreover, *P*_*l*_ with $\sum_{l=1}^{c}p_{l}=1$, is the mixing proportion of cluster *l*. The mixing proportion *p*_*l*_ is defined as the proportion of individuals that belong to the *l*-th cluster. The joint log-likelihood function for *n* independent individual vectors is defined as follows:

$$\ln L=\sum_{i=1}^{n}\ln f(Y_{i}).$$ (2)

### Clustering algorithm for missing values

Most reports [23–25] consider only complete datasets (without missing values) for Gaussian mixture clustering. Indeed, it is challenging to estimate missing values accurately. An urgent problem concerns how to provide the optimal number of clusters *c* and how to cluster *n* individuals into the *c* clusters precisely. Therefore, we propose a missing-value imputation algorithm as follows.

Suppose that there are *r* missing values in Y_*i*_. Thus, *y*_*i*1_, *y*_*i*2_,…, *y*_*ir*_ are missing. The Y_*i*_, μ_*l*_, and S_*l*_ are then divided into

$$Y_{i}=\left(Y_{i(1)},\;Y_{i(2)}\right),\;\mu_{l}=\left(\mu_{l(1)},\;\mu_{l(2)}\right),\;\mathrm{and}\;S_{l}=\left[\begin{array}{cc} S_{l}{}_{11} & S_{l}{}_{12}\\ S_{l}{}_{21} & S_{l}{}_{22} \end{array}\right],$$

where

$$Y_{i(1)}=\left(y_{i1},y_{i2},\ldots ,y_{ir}\right),\;Y_{i(2)}=\left(y_{i(r+1)},y_{i(r+2)},\ldots ,y_{ik}\right),$$

$$\mu_{l(1)}=\left(\mu_{l1},\mu_{l2},\ldots ,\mu_{lr}\right),\;\mu_{l(2)}=\left(\mu_{l(r+1)},\mu_{l(r+2)},\ldots ,\mu_{lk}\right),$$

$$S_{l}{}_{11}=\left[\begin{array}{cccc} s_{l1}^{2} & s_{l12} & \cdots & s_{l1r}\\ s_{l21} & s_{l2}^{2} & \cdots & s_{l2r}\\ \vdots & \vdots & \ddots & \vdots \\ s_{lr1} & s_{lr2} & \cdots & s_{lr}^{2} \end{array}\right],\;S_{l}{}_{12}=\left[\begin{array}{cccc} s_{l1(r+1)} & s_{l1(r+2)} & \cdots & s_{l1k}\\ s_{l2(r+1)} & s_{l2(r+2)} & \cdots & s_{l2k}\\ \vdots & \vdots & \ddots & \vdots \\ s_{lr(r+1)} & s_{lr(r+2)} & \cdots & s_{lrk} \end{array}\right],$$

$$S_{l}{}_{21}=\left[\begin{array}{cccc} s_{l(r+1)1} & s_{l(r+1)2} & \cdots & s_{l(r+1)r}\\ s_{l(r+2)1} & s_{l(r+2)2} & \cdots & s_{l(r+2)r}\\ \vdots & \vdots & \ddots & \vdots \\ s_{lk1} & s_{lk2} & \cdots & s_{lkr} \end{array}\right],\;\text{and}\;S_{l}{}_{22}=\left[\begin{array}{cccc} s_{l(r+1)}^{2} & s_{l(r+1)(r+2)} & \cdots & s_{l(r+1)k}\\ s_{l(r+2)(r+1)} & s_{l(r+2)}^{2} & \cdots & s_{l(r+2)k}\\ \vdots & \vdots & \ddots & \vdots \\ s_{lk(r+1)} & s_{lk(r+2)} & \cdots & s_{lk}^{2} \end{array}\right].$$

Suppose further that Y_*i*_ is from the *l*-th *k*-dimensional Gaussian distribution and that Y_*i*(2)_ is known. The conditional expectation of Y_*i*(1)_, which belongs to the *l*-th cluster, is derived as follows:

$$E_{l}(Y_{i(1)}|Y_{i(2)})=\mu_{l(1)}+S_{l}{}_{12}S_{l22}^{-1}(Y_{i(2)}-\mu_{l(2)}).$$ (3)

We provide the initial value for μ_*l*(1)_, S_*l*11_, and S_*l*12_. Then, based on the criterion of the minimum mean-square deviations from Eq (3), the conditional expectation E_*l*_(Y_*i*(1)_|Y_*i*(2)_) can be calculated, representing the best predicted function for Y_*i*(1)_ [26]. Under the mixture distribution of the individual vector Y_*i*_, the conditional expectation for Y_*i*(1)_ based on Y_*i*(2)_ is

$$E(Y_{i(1)}|Y_{i(2)})=\sum_{l=1}^{c}p_{l}\;E_{l}(Y_{i(1)}|Y_{i(2)}),$$ (4)

where E(Y_*i*(1)_|Y_*i*(2)_) denotes the estimators for the missing values Y_*i*(1)_. Subsequently, a “pseudo-complete” dataset can be constructed.

### Iterative clustering algorithm

The proposed model-based clustering algorithm for datasets with missing values assigns each individual to one of the *c* clusters with a certain probability. We define this probability as *p*_*li*_, which is that of the *i*-th individual belonging to the *l*-th cluster. An individual is assigned to the *l*-th cluster if *p*_*li*_ is greater than a certain pre-determined threshold. The probability can be calculated using the expectation-maximization (EM) algorithm [27] to achieve the maximum likelihood for the objective function derived with Eq (2). The EM algorithm begins with some initial parameters that are set in advance. Then, each parameter is iteratively updated in the algorithm until convergence to a minimizer is reached. An EM iteration includes the following steps:

1. Initialize the prior probabilities of the cluster assignment and the cluster parameters:
  $$q^{\left(t\right)}=\left(p_{1}^{\left(t\right)},\ldots ,p_{c}^{\left(t\right)},\mu_{1}^{\left(t\right)},\ldots ,\mu_{c}^{\left(t\right)},S_{1}^{\left(t\right)},\ldots ,S_{c}^{\left(t\right)}\right).$$ (5)
2. Calculate the missing values $Y_{i(1)}^{\left(t\right)}$ using Eqs (3) and (4) to generate the “pseudo-complete” dataset for $Y_{i}^{\left(t\right)}$.
3. Update the posterior probabilities of the cluster assignment:
  $$p_{li}^{\left(t\right)}=\frac{p_{l}^{\left(t\right)}f_{l}^{\left(t\right)}(Y_{i}^{\left(t\right)})}{\sum_{h=1}^{c}p_{h}^{\left(t\right)}f_{h}^{\left(t\right)}(Y_{h}^{\left(t\right)})}.$$ (6)
4. Update the cluster proportions, mean vectors, and variance-covariance matrices:
  $$p_{l}^{\left(t+1\right)}=\sum_{i=1}^{n}p_{li}^{\left(t\right)}/n;$$ (7)
  $$\mu_{l}^{\left(t+1\right)}=\sum_{i=1}^{n}p_{li}^{\left(t\right)}Y_{i}^{\left(t\right)}/\sum_{i=1}^{n}p_{li}^{\left(t\right)};$$ (8)
  $$S_{l}^{\left(t+1\right)}=\sum_{i=1}^{n}p_{li}^{\left(t\right)}\left[{\left(Y_{i}^{\left(t\right)}-\mu_{l}^{\left(t+1\right)}\right)}^{\text{T}}\left(Y_{i}^{\left(t\right)}-\mu_{l}^{\left(t+1\right)}\right)\right]/\sum_{i=1}^{n}p_{li}^{\left(t\right)}.$$ (9)
5. Repeat steps (1)–(4) until convergence is reached.

The number of clusters *c* can also be treated as an unknown parameter and inferred by the BIC or AIC tests. The BIC is derived as follows:

$$\text{BIC}=2\;\ln\;L(\hat{\theta })-q\ln(n),$$ (10)

where *q* = *c*(*k*+1)(*k*+2)/2−1 is the number of independent parameters to be estimated in the model, $\text{L}(\hat{\theta })$ is the likelihood of $\hat{\theta }$—i.e., the vector for the maximum likelihood estimation of the parameters—and *n* is the size of the dataset. The number of clusters *c* is determined by the maximum BIC value.

## Results

### Simulation analysis

A simulation was designed to evaluate the feasibility and accuracy of the proposed missing-values imputation algorithm. The individuals belonging to each cluster were known exactly and without subjective errors. Indeed, when analyzing real data, the misjudgment rate (MR) may reflect confounding errors in the experiment and the subjective errors. Without loss of generality, we simulated datasets with a two-dimensional Gaussian distribution and some missing values. All simulations were performed using statistical SAS (version 9.3; SAS/IML) software.

#### Design of the simulation

Suppose that there are 500 individuals derived from one of two (two-dimensional) Gaussian populations, denoted by *MVN* (**μ**_1_,**S**_1_) and *MVN* (**μ**_2_,**S**_2_), from which 10% of the individuals have at least one variable randomly removed. The entire dataset contains 1000 individuals. Each individual has two variables, and 100 individuals have at least one variable missing. The cluster’s mean vectors are denoted by **μ**_1_ and **μ**_2_, which were simulated at three different levels: *A*_1_: **μ**_1_ = (0, 0), **μ**_2_ = (2.5, 2.5), *A*_2_: **μ**_1_ = (0, 0), **μ**_2_ = (2.0, 2.0), and *A*_3_: **μ**_1_ = (0, 0), **μ**_2_ = (1.5, 1.5). The variance-covariance matrices are denoted by **S**_1_ and **S**_2_ (without loss of generality, supposing that **S**_1_ = **S**_2_ = **S**), and these were set at four levels: $B_{1}:\;S=0.25\;\left[\begin{array}{cc} 1 & 0.6\\ 0.6 & 1 \end{array}\right]$, $B_{2}:\;S=0.5\;\left[\begin{array}{cc} 1 & 0.6\\ 0.6 & 1 \end{array}\right]$, $B_{3}:\;S=0.75\;\left[\begin{array}{cc} 1 & 0.6\\ 0.6 & 1 \end{array}\right]$, and $B_{4}:\;S=\left[\begin{array}{cc} 1 & 0.6\\ 0.6 & 1 \end{array}\right]$. The total number of treatment combinations (datasets) is therefore 3 × 4 = 12, that is, A_1_B_1_, A_1_B_2_, A_1_B_3_, A_1_B_4_, A_2_B_1_, A_2_B_2_, A_2_B_3_, A_2_B_4_, A_3_B_1_, A_3_B_2_, A_3_B_3_, and A_3_B_4._ Twenty replicated simulations were conducted for each of the twelve scenarios.

Our missing-value imputation cluster algorithm (denoted by M-3, generating a “pseudo-complete” dataset) was compared with three other cluster algorithms: a cluster algorithm using the complete dataset (denoted by M-1, i.e., the above simulation dataset without any missing values); a cluster algorithm using a dataset from which all individuals with missing variables are deleted (denoted by M-2, i.e., a cropped dataset); and a cluster algorithm using missing-value imputation by mean replacement in which the missing values are replaced by the mean value of the whole dataset (denoted by M-4, another “pseudo-complete” dataset). Thus, we used these four algorithms to cluster simulated datasets. These algorithms are all based on the multivariate Gaussian mixture model, using the maximum likelihood estimation with the EM algorithm. As such, the clustering results could be fairly compared. We evaluated M-1, M-2, M-3, and M-4 using the following metrics: (1) the average convergence rate; (2) the accuracy and precision of their respective parameter estimates; and (3) the total misjudgment rate (MR), where MR is the ratio of all misjudged individuals to the total number of individuals in the 20 replicates and MR = 1 - accuracy. A chi-square test and multiple comparisons of Scheffé’s Confidence Interval were used to test the differences in MR among the four algorithms.

#### Simulation results

The average parameter estimates include the mean vectors (± se), variance-covariance matrices (± se), the likelihood value of the maximum-likelihood function, and the total MR for the four clustering algorithms. The results are presented in Tables 1–3. Our proposed missing-value imputation clustering algorithm (M-3) outperformed the other two algorithms (M-2 and M-4) in terms of the convergence rate for most of the simulation datasets. Indeed, its superiority was most obvious when the mean vectors of two clusters were near each other and the variance-covariance of each cluster was more disperse, such as with datasets A_2_B_4_, A_3_B_3_, and A_3_B_4_. Moreover, the proposed algorithm resulted in the most accurate and precise parameter estimations, closely approximating the true values.

**Table 1:** Average parameter estimates under 4 different simulation datasets *A*_1_*B*_1_-*A*_1_*B*_4_ in 20 replicates.

Treatment		Iterative time	Likelihood value	Probability estimate		Mean vector estimate				Covariance matrix estimate				MR (%)
				${\hat{P}}_{1}$	${\hat{P}}_{2}$	${\hat{\mu }}_{1}$ ± se		${\hat{\mu }}_{2}$ ± se		${\hat{S}}_{1}$ ± se		${\hat{S}}_{2}$ ± se		
*A*_1_*B*_1_	M-1	51	-1876.32	0.50	0.50	0.00±0.01	0.00±0.01	2.49±0.07	2.50±0.06	0.25±0.04	0.15±0.03	0.25±0.04	0.15±0.02	0.20^*a*^
										0.15±0.03	0.26±0.04	0.15±0.02	0.25±0.04	
	M-2	58	-1864.94	0.50	0.50	0.00±0.01	-0.01±0.02	2.47±0.08	2.53±0.07	0.24±0.05	0.14±0.03	0.25±0.03	0.15±0.02	0.30^*a*^
										0.14±0.03	0.26±0.04	0.15±0.02	0.24±0.04	
	M-3	64	-1897.05	0.50	0.50	0.00±0.02	0.00±0.01	2.49±0.07	2.50±0.06	0.24±0.04	0.14±0.02	0.24±0.04	0.14±0.04	0.20^*a*^
										0.14±0.02	0.26±0.03	0.14±0.04	0.25±0.04	
	M-4	55	-1800.38	0.49	0.49	-0.01±0.03	0.00±0.02	2.46±0.08	2.46±0.09	0.22±0.07	0.13±0.04	0.23±0.06	0.14±0.05	1.50^*b*^
										0.13±0.04	0.23±0.06	0.14±0.05	0.21±0.07	
*A*_1_*B*_2_	M-1	70	-2578.47	0.50	0.50	0.01±0.03	0.00±0.04	2.47±0.14	2.47±0.14	0.48±0.06	0.28±0.04	0.47±0.07	0.28±0.04	2.40^*a*^
										0.28±0.04	0.53±0.08	0.28±0.04	0.48±0.06	
	M-2	80	-2362.69	0.50	0.50	-0.01±0.04	0.01±0.04	2.52±0.15	2.48±0.16	0.48±0.07	0.32±0.04	0.47±0.07	0.26±0.06	2.80^*a*^
										0.32±0.04	0.54±0.08	0.26±0.06	0.46±0.08	
	M-3	87	-2591.61	0.50	0.50	-0.01±0.04	0.01±0.03	2.47±0.14	2.46±0.16	0.47±0.06	0.27±0.05	0.46±0.07	0.28±0.04	2.60^*a*^
										0.27±0.05	0.53±0.07	0.28±0.04	0.48±0.06	
	M-4	93	-2653.93	0.48	0.48	0.02±0.09	0.01±0.08	2.43±0.26	2.44±0.23	0.42±0.14	0.25±0.11	0.44±0.12	0.25±0.11	5.64^*b*^
										0.25±0.11	0.43±0.13	0.25±0.11	0.44±0.12	
*A*_1_*B*_3_	M-1	107	-2837.52	0.49	0.51	0.02±0.07	-0.01±0.08	2.47±0.21	2.47±0.21	0.68±0.16	0.42±0.10	0.71±0.14	0.43±0.09	5.90 ^*ab*^
										0.42±0.10	0.78±0.15	0.43±0.09	0.75±0.14	
	M-2	142	-2569.69	0.50	0.50	-0.02±0.06	0.01±0.06	2.48±0.20	2.52±0.19	0.73±0.16	0.47±0.08	0.69±0.16	0.40±0.09	5.45^*a*^
										0.47±0.08	0.81±0.15	0.40±0.09	0.67±0.17	
	M-3	134	-2822.26	0.49	0.51	-0.01±0.07	-0.02±0.06	2.45±0.21	2.46±0.20	0.65±0.17	0.39±0.08	0.73±0.14	0.42±0.07	6.12^*ab*^
										0.39±0.08	0.76±0.12	0.42±0.07	0.68±0.15	
	M-4	138	-2859.62	0.45	0.55	-0.05±0.15	0.04±0.12	2.40±0.37	2.43±0.32	0.63±0.21	0.40±0.16	0.70±0.19	0.39±0.11	8.94^*b*^
										0.40±0.16	0.60±0.20	0.39±0.11	0.71±0.19	
*A*_1_*B*_4_	M-1	180	-3211.91	0.49	0.52	-0.05±0.10	-0.03±0.12	2.40±0.25	2.42±0.28	0.86±0.24	0.51±0.17	0.94±0.24	0.59±0.15	8.50^*a*^
										0.51±0.17	1.05±0.26	0.59±0.15	1.02±0.23	
	M-2	214	-2852.78	0.48	0.52	-0.04±0.10	-0.06±0.14	2.38±0.30	2.37±0.32	0.84±0.25	0.55±0.16	0.97±0.19	0.56±0.16	9.20^*a*^
										0.55±0.16	1.05±0.26	0.56±0.16	0.94±0.25	
	M-3	215	-3100.00	0.48	0.52	-0.05±0.12	-0.06±0.13	2.39±0.26	2.38±0.30	0.84±0.23	0.48±0.19	0.90±0.25	0.57±0.14	8.70^*a*^
										0.48±0.19	1.02±0.21	0.57±0.14	0.99±0.20	
	M-4	267	-3289.32	0.43	0.57	-0.07±0.20	-0.08±0.25	2.17±0.48	2.22±0.52	0.77±0.32	0.43±0.28	0.82±0.27	0.48±0.15	18.90^*b*^
										0.43±0.28	1.09±0.23	0.48±0.15	1.07±0.25	

M-1 indicates a complete data clustering algorithm; M-2 indicates a missing-data-deleted clustering algorithm; M-3 indicates our missing-value imputation clustering algorithm

M-4 indicates the clustering algorithm for missing-value imputation by mean replacement. These four algorithms are based on the multivariate Gaussian mixture model.

^*a*,*b*^ indicates the multiple comparisons of the differences in MR among the algorithms M-1, M-2, M-3, and M-4: having the different letters indicate that there is statistical significance between these two groups (P<0.05), and vice versa.

**Table 2:** Average parameter estimates under 4 different simulation datasets *A*_2_*B*_1_-*A*_2_*B*_4_ in 20 replicates.

Treatment		Iterative time	Likelihood value	Probability estimate		Mean vector estimate				Covariance matrix estimate				MR (%)
				${\hat{P}}_{1}$	${\hat{P}}_{2}$	${\hat{\mu }}_{1}$ ± se		${\hat{\mu }}_{2}$ ± se		${\hat{S}}_{1}$ ± se		${\hat{S}}_{2}$ ± se		
*A*_2_*B*_1_	M-1	81	-1903.64	0.50	0.50	-0.01±0.04	0.00±0.03	2.00±0.12	2.00±0.13	0.25±0.05	0.15±0.04	0.24±0.04	0.14±0.04	1.64^*a*^
										0.15±0.04	0.25±0.05	0.14±0.04	0.24±0.05	
	M-2	117	-1696.53	0.51	0.49	0.01±0.04	-0.01±0.04	2.00±0.14	1.99±0.13	0.24±0.05	0.15±0.03	0.25±0.05	0.14±0.04	1.86^*a*^
										0.15±0.03	0.26±0.04	0.14±0.04	0.24±0.06	
	M-3	92	-1885.63	0.50	0.50	-0.01±0.04	0.01±0.04	1.99±0.14	1.99±0.12	0.23±0.05	0.14±0.04	0.23±0.06	0.15±0.04	1.60^*a*^
										0.14±0.04	0.26±0.04	0.15±0.04	0.24±0.05	
	M-4	126	-2008.47	0.48	0.52	-0.02±0.07	0.01±0.06	1.95±0.19	1.96±0.20	0.22±0.10	0.12±0.07	0.21±0.10	0.11±0.05	2.26^*a*^
										0.12±0.07	0.27±0.12	0.11±0.05	0.27±0.07	
*A*_2_*B*_2_	M-1	120	-2372.14	0.50	0.50	-0.02±0.07	-0.01±0.06	2.01±0.16	1.97±0.17	0.45±0.13	0.27±0.09	0.46±0.14	0.28±0.10	5.60^*a*^
										0.27±0.09	0.52±0.11	0.28±0.10	0.50±0.14	
	M-2	179	-2128.49	0.51	0.49	-0.02±0.08	0.02±0.06	1.95±0.18	1.96±0.18	0.48±0.11	0.27±0.08	0.47±0.14	0.25±0.09	6.30^*ab*^
										0.27±0.08	0.53±0.13	0.25±0.09	0.45±0.13	
	M-3	135	-2462.73	0.51	0.49	0.01±0.07	0.02±0.06	1.95±0.19	1.97±0.17	0.46±0.11	0.28±0.08	0.44±0.09	0.26±0.09	5.40^*a*^
										0.28±0.08	0.53±0.10	0.26±0.09	0.46±0.08	
	M-4	266	-2410.50	0.45	0.55	-0.03±0.10	0.02±0.11	1.91±0.28	1.92±0.29	0.44±0.17	0.25±0.10	0.44±0.18	0.24±0.11	9.00 ^*b*^
										0.25±0.10	0.54±0.16	0.24±0.11	0.43±0.19	
*A*_2_*B*_3_	M-1	219	-2699.35	0.48	0.52	-0.05±0.08	-0.03±0.08	1.92±0.27	1.94±0.24	0.67±0.20	0.40±0.10	0.75±0.19	0.42±0.11	10.90^*a*^
										0.40±0.10	0.78±0.21	0.42±0.11	0.78±0.19	
	M-2	387	-2489.38	0.47	0.53	-0.07±0.09	0.05±0.10	1.90±0.27	1.90±0.28	0.64±0.25	0.38±0.13	0.72±0.22	0.41±0.12	11.40^*a*^
										0.38±0.13	0.73±0.24	0.41±0.12	0.79±0.21	
	M-3	242	-2709.34	0.47	0.53	-0.07±0.10	-0.05±0.10	1.90±0.28	1.90±0.30	0.66±0.18	0.38±0.09	0.76±0.20	0.47±0.11	11.00^*a*^
										0.42±0.09	0.77±0.18	0.47±0.11	0.75±0.20	
	M-4	450	-2646.83	0.42	0.58	-0.14±0.20	0.12±0.18	1.89±0.41	1.88±0.45	0.64±0.29	0.38±0.17	0.72±0.27	0.40±0.15	16.96^*b*^
										0.38±0.17	0.78±0.27	0.40±0.15	0.77±0.23	
*A*_2_*B*_4_	M-1	360	-3014.36	0.45	0.55	-0.11±0.16	0.09±0.17	1.85±0.34	1.80±0.32	0.86±0.26	0.52±0.18	1.01±0.23	0.63±0.17	12.80^*a*^
										0.52±0.18	1.04±0.25	0.63±0.17	1.05±0.23	
	M-2	486	-2677.25	0.43	0.57	-0.15±0.25	-0.15±0.25	1.84±0.35	1.82±0.32	0.82±0.30	0.51±0.19	1.04±0.29	0.61±0.18	18.00^*b*^
										0.51±0.19	0.94±0.25	0.61±0.18	1.01±0.29	
	M-3	342	-2907.18	0.44	0.56	-0.10±0.17	0.08±0.14	1.86±0.34	1.82±0.32	0.87±0.27	0.53±0.18	0.92±0.28	0.62±0.17	12.30^*a*^
										0.53±0.18	1.03±0.25	0.62±0.17	1.03±0.29	
	M-4	440	-2977.46	0.40	0.60	-0.20±0.35	0.19±0.33	1.82±0.57	1.81±0.62	0.76±0.41	0.51±0.27	1.05±0.33	0.65±0.27	21.80^*c*^
										0.51±0.27	0.90±0.39	0.65±0.27	1.07±0.34	

M-1 indicates a complete data clustering algorithm; M-2 indicates a missing-data-deleted clustering algorithm; M-3 indicates our missing-value imputation clustering algorithm

M-4 indicates the clustering algorithm for missing-value imputation by mean replacement. These four algorithms are based on the multivariate Gaussian mixture model.

^*a*,*b*,*c*^ indicates the multiple comparisons of the differences in MR among the algorithms M-1, M-2, M-3, and M-4: having the different letters indicate that there is statistical significance between these two groups (P<0.05), and vice versa.

**Table 3:** Average parameter estimates under 12 different simulation datasets *A*_3_*B*_1_-*A*_3_*B*_4_ in 20 replicates.

Treatment		Iterative time	Likelihood value	Probability estimate		Mean vector estimate				Covariance matrix estimate				MR (%)
				${\hat{P}}_{1}$	${\hat{P}}_{2}$	${\hat{\mu }}_{1}$ ± se		${\hat{\mu }}_{2}$ ± se		${\hat{S}}_{1}$ ± se		${\hat{S}}_{2}$ ± se		
*A*_3_*B*_1_	M-1	129	-1816.34	0.50	0.50	0.00±0.06	0.01±0.06	1.47±0.15	1.48±0.14	0.23±0.08	0.13±0.05	0.23±0.09	0.14±0.06	4.90^*a*^
										0.13±0.05	0.26±0.09	0.14±0.06	0.25±0.10	
	M-2	337	-1637.81	0.51	0.49	0.01±0.07	0.01±0.06	1.53±0.15	1.47±0.16	0.23±0.08	0.14±0.06	0.23±0.10	0.12±0.06	5.35^*a*^
										0.14±0.06	0.27±0.10	0.12±0.06	0.22±0.09	
	M-3	134	-1762.64	0.50	0.50	-0.01±0.07	0.00±0.06	1.46±0.16	1.46±0.16	0.21±0.08	0.13±0.05	0.21±0.09	0.14±0.05	5.00^*a*^
										0.13±0.05	0.26±0.08	0.14±0.05	0.25±0.08	
	M-4	359	-1800.32	0.52	0.48	-0.02±0.09	0.03±0.08	1.44±0.25	1.45±0.26	0.20±0.14	0.13±0.07	0.21±0.13	0.14±0.06	9.20^*b*^
										0.13±0.07	0.24±0.13	0.14±0.06	0.25±0.13	
*A*_3_*B*_2_	M-1	312	-2322.43	0.45	0.55	-0.06±0.10	-0.03±0.07	1.43±0.26	1.45±0.25	0.45±0.23	0.22±0.11	0.45±0.23	0.32±0.09	11.50^*a*^
										0.22±0.11	0.55±0.23	0.32±0.09	0.53±0.22	
	M-2	444	-2102.31	0.45	0.55	-0.08±0.11	0.07±0.10	1.41±0.27	1.42±0.26	0.41±0.25	0.23±0.13	0.45±0.24	0.30±0.10	13.45^*ab*^
										0.23±0.13	0.47±0.25	0.30±0.10	0.54±0.23	
	M-3	300	-2296.72	0.46	0.54	-0.06±0.10	-0.03±0.08	1.42±0.26	1.43±0.25	0.44±0.23	0.25±0.10	0.46±0.22	0.30±0.09	12.00^*a*^
										0.25±0.10	0.54±0.22	0.30±0.09	0.54±0.21	
	M-4	489	-2358.99	0.43	0.57	-0.09±0.25	0.07±0.26	1.39±0.33	1.40±0.35	0.41±0.28	0.25±0.14	0.43±0.29	0.30±0.13	16.50 ^*b*^
										0.25±0.14	0.54±0.29	0.30±0.13	0.52±0.28	
*A*_3_*B*_3_	M-1	584	-2586.53	0.42	0.60	-0.15±0.28	-0.14±0.26	1.38±0.46	1.40±0.47	0.68±0.32	0.49±0.18	0.80±0.31	0.49±0.16	18.00^*a*^
										0.49±0.18	0.72±0.31	0.49±0.16	0.78±0.30	
	M-2	603	-2364.52	0.39	0.63	-0.19±0.35	-0.18±0.33	1.38±0.52	1.37±0.50	0.64±0.35	0.48±0.17	0.81±0.32	0.46±0.18	23.30 ^*b*^
										0.48±0.17	0.65±0.34	0.46±0.18	0.75±0.32	
	M-3	310	-2578.77	0.44	0.58	-0.10±0.25	-0.10±0.26	1.40±0.45	1.39±0.45	0.65±0.31	0.44±0.17	0.74±0.29	0.47±0.15	18.00^*a*^
										0.44±0.17	0.78±0.32	0.47±0.15	0.78±0.29	
	M-4	590	-2600.62	0.37	0.63	-0.18±0.38	-0.17±0.41	1.38±0.55	1.37±0.54	0.63±0.39	0.42±0.25	0.82±0.39	0.48±0.21	25.00^*b*^
										0.42±0.25	0.68±0.40	0.48±0.21	0.75±0.39	
*A*_3_*B*_4_	M-1	645	-3013.37	0.35	0.65	-0.13±0.46	-0.17±0.38	1.30±0.72	1.30±0.70	0.92±0.38	0.50±0.20	1.14±0.40	0.68±0.24	28.00^*b*^
										0.50±0.20	1.08±0.34	0.68±0.24	0.97±0.39	
	M-2	690	-2633.70	0.20	0.80	-0.22±0.74	-0.15±0.62	1.00±0.99	1.20±0.98	0.70±0.57	0.38±0.40	1.26±0.62	1.01±0.38	48.32^*c*^
										0.38±0.40	0.79±0.51	1.01±0.38	1.23±0.58	
	M-3	520	-2892.21	0.40	0.60	-0.11±0.34	0.15±0.31	1.34±0.66	1.37±0.63	0.93±0.36	0.62±0.18	1.07±0.37	0.69±0.20	21.30^*a*^
										0.62±0.18	1.07±0.39	0.69±0.20	0.96±0.41	
	M-4	835	-2901.33	0.10	0.90	-0.58±0.74	-0.55±0.80	0.90±1.04	0.97±1.07	0.61±0.72	0.21±0.58	1.26±0.77	0.89±0.47	56.00^*d*^
										0.21±0.58	0.62±0.74	0.89±0.47	1.23±0.77	

M-1 indicates a complete data clustering algorithm; M-2 indicates a missing-data-deleted clustering algorithm; M-3 indicates our missing-value imputation clustering algorithm

M-4 indicates the clustering algorithm for missing-value imputation by mean replacement. These four algorithms are based on the multivariate Gaussian mixture model.

^*a*,*b*,*c*,*d*^ indicates the multiple comparisons of the differences in MR among the algorithms M-1, M-2, M-3, and M-4: having the different letters indicate that there is statistical significance between these two groups (P<0.05), and vice versa.

Thus, our proposed missing-value imputation clustering algorithm correctly clusters individuals with missing variables. The total MR resulting from the proposed algorithm (M-3) demonstrates that its performance is superior to that of M-4 in all simulation trials, with significant statistical difference (p < 0.05), and superior to that of M-2 in simulation trials A_2_B_4_, A_3_B_3_, and A_3_B_4_, with significant statistical difference (p < 0.05). Moreover, the clustering accuracy of our proposed algorithm was comparable to that of the complete dataset (M-1) (p > 0.05). In general, the more similar the mean vectors of two clusters or the more disperse their variance-covariance, the higher the MR of all clustering methods and the better the performance of our proposed algorithm compared to M-2 and M-4. Interestingly, however, our algorithm performed well in such cases. For example, in scenario A_3_B_4_, the speed of convergence with M-2 and M-4 was lower in both cases than for other scenarios and had a total MR of 48.32% and 56.00%, respectively, whereas the total MR with our proposed algorithm M-3 was 21.30%. In fact, this result was slightly better than M-1, which resulted in an MR of 28.00%.

### Real data analysis

#### Iris dataset

Fisher’s Iris dataset (Fisher, 1936) [12] is perhaps the best-known database for comparing clustering algorithms. The dataset contains three clusters with 50 individuals each, where each cluster is a species of the genus *Iris*: namely, *Iris setosa*, *Iris versicolor*, and *Iris virginica*. These three species are thus segregated. The dataset contains four variables: sepal length, sepal width, petal length, and petal width. Fisher’s Iris dataset is available on the Internet (http://archive.ics.uci.edu/ml/datasets/Iris). We randomly removed one of the four variables in 10% of the individual observations of *Iris* data; the resulting dataset constituted a dataset with missing values. Table 4 lists the clustering results from five clustering algorithms: the above four clustering algorithms [the clustering algorithm with a complete dataset (M-1), the clustering algorithm in which individuals with missing variables are deleted (M-2), our proposed missing-value imputation algorithm (M-3), and the clustering algorithm for missing-value imputation by mean replacement (M-4)] and the FCM clustering method applied to the dataset with data missing from 10% of the individuals. In the M-4 and FCM methods, the missing values are replaced by the mean of the whole dataset. The superiority of our proposed method is obvious insofar as M-3 resulted in the lowest MR of the five algorithms. Because of Gaussian mixture distribution of the dataset, our algorithm makes full use of the known data based on the Gaussian mixture model to estimate missing values, the accuracy of our method was higher than those of the FCM method and the M-2 and M-4 algorithms, and it closely approximated the accuracy obtained using the complete-dataset clustering algorithm (M-1). Moreover, the model-based algorithms (M-1, M-2, M-3, and M-4) resulted in the highest BIC values in the three clusters, validating the effectiveness of using the BIC to determine the number of clusters.

**Table 4:** The comparison among four algorithms and FCM method for Fisher’s Iris dataset.

Method	n	Misjudgment rate (MR)	Iterative time	BIC value in three clusters
M-1	150	3 (2.0%)	37	-307.848
M-2	135	6 (4.4%)	43	-273.539
M-3	150	3 (2.0%)	36	-305.211
M-4	150	7 (4.7%)	49	-307.382
FCM	150	5 (3.3%)		

M-1 indicates the complete data clustering algorithm; M-2 indicates missing data deleted clustering algorithm; M-3 indicates our missing-value imputation clustering algorithm; M-4 indicates clustering algorithm for missing-value imputation by mean replacement; FCM indicates Fuzzy C-Means clustering.

#### Yeast Cell-cycle dataset

The Yeast Cell-cycle dataset [28] shows the fluctuation in the expression levels of the whole genome (6220 genes) over 17 time points taken at 10-minute intervals, covering nearly two cell cycles. The entire raw dataset is available at http://genome-www.stanford.edu/cellcycle/. Among the effectively expressed genes, 416 genes were found to have consistent periodic changes in expression level. Yeung et al. picked out 384 genes over 15 time points that reached their peaks at five (*C* = 5) cell phases on the basis of the size of the buds, after which five non-intersecting clusters were classified [29]. These 384 gene expressions, a subset with a moderate sample size, are available at http://www.cs.washington.edu/homes/kayee/model. All 384 genes were assigned to one of the five clusters by the original researchers [28,29]. Therefore, we can use this dataset to test the performance of the clustering algorithms introduced here and compare them with the performance of existing methods. We randomly removed one of the 15 variable values in 10% of the genes to create a dataset with missing values. Table 5 lists the clustering results from five clustering algorithms: the above four model-based clustering algorithms (M-1, M-2, M-3, and M-4) and the FCM clustering method. Further validating the results from Fisher’s Iris dataset, we found that our missing-value imputation algorithm had the lowest MR. In addition, because our missing-value imputation based on the Gaussian mixture distribution is compatible with the Yeast Cell-cycle dataset, the accuracy of our method was significantly higher than that of the FCM method, and it closely approximated the accuracy from using the complete-dataset clustering algorithm (M-1).

**Table 5:** The comparison among four algorithms and FCM method for Yeast Cell-cycle dataset.

Method	n	Misjudgment rate (MR)	Iterative time	BIC value in five clusters
M-1	384	65 (16.9%)	1265	-6472.448
M-2	346	62 (17.9%)	1340	-5892.953
M-3	384	62 (16.1%)	1260	-6316.730
M-4	384	78 (20.3%)	1307	-6490.706
FCM	384	80 (20.8%)		

M-1 indicates the complete data clustering algorithm; M-2 indicates missing data deleted clustering algorithm; M-3 indicates our missing-value imputation clustering algorithm; M-4 indicates the clustering algorithm for missing-value imputation by mean replacement; FCM indicates Fuzzy C-Means clustering.

#### CIFAR-10 images dataset

The CIFAR-10 dataset [30] contains 60,000 32 × 32 color images (3072 variables) divided into ten classes, which represent different kinds of pictures. The classes are designed to be completely mutually exclusive. For example, neither automobile pictures nor truck pictures contain images of pickup trucks. Having a large sample size, each class contains 5000 training images and 1000 test images. The entire dataset was used to test the performance of test images according to training images in supervised classification, and it is available at http://www.cs.toronto.edu/~kriz/cifar.html. We used the training images to test the performance of our unsupervised clustering algorithm and compared it to existing clustering algorithms. We randomly removed 10 of the 3072 variable values in 10% of the color images to create a dataset with missing values.

Table 6 lists the clustering results from five clustering algorithms: the above four model-based clustering algorithms (M-1, M-2, M-3, and M-4) and the FCM clustering method. We found that our missing-value imputation algorithm had a lower total MR than algorithms M-2 and M-4. The accuracy of our method closely approximated the accuracy obtained using the clustering algorithm with complete data (M-1) but was lower than that of the FCM method. (It should be noted that most clusters of the CIFAR-10 data have deviants from the normal distribution.) Thus, the performance of our model-based algorithm does not offer clustering that is more accurate than the FCM method. Moreover, the speed of computations was much lower in algorithms M-1, M-2, M-3, and M-4.

**Table 6:** The comparison among four algorithms and FCM method for CIFAR-10 dataset.

Method	MR										
	total	Cluster 1	Cluster 2	Cluster 3	Cluster 4	Cluster 5	Cluster 6	Cluster 7	Cluster 8	Cluster 9	Cluster 10
M-1	39.9%	38.2%	41.4%	35.6%	43.5%	40.5%	37.9%	38.5%	42.1%	35.4%	45.6%
M-2	42.4%	42.6%	40.8%	35.2%	45.8%	42.0%	46.3%	40.4%	46.7%	38.4%	45.4%
M-3	39.8%	42.0%	41.0%	36.2%	39.6%	39.7%	38.9%	38.0%	43.2%	34.2%	45.0%
M-4	44.0%	45.3%	43.6%	37.4%	45.9%	43.7%	48.5%	42.8%	47.9%	41.3%	43.9%
FCM	35.9%	35.8%	35.6%	35.5%	36.7%	34.1%	37.6%	32.6%	37.6%	35.7%	37.8%

M-1 indicates the complete data clustering algorithm; M-2 indicates missing data deleted clustering algorithm; M-3 indicates our missing-value imputation clustering algorithm; M-4 indicates clustering algorithm for missing-value imputation by mean replacement; FCM indicates Fuzzy C-Means clustering.

## Discussion

Missing values are unavoidable in experimental data and data from field investigations [31]. Many imputation methods [16–20] have been proposed, such as the mean-substitution method, the regression imputation method, the EM estimation method, and the FCM method. Clustering is a widely used statistical method, especially in gene expression profiles. Most classical clustering methods, such as K-means clustering and hierarchical clustering, are unable to deal with missing values. Therefore, we developed a model-based clustering algorithm for imputing missing values. The proposed method utilizes information from existing data, and it is based on the Gaussian mixture model. With our imputation algorithm, missing values are estimated by calculating the conditional expectation, from which a “pseudo-complete” dataset can be generated and used for clustering.

The performance of our proposed algorithm was tested and compared with other algorithms using both real and simulated data. We found that our proposed method outperformed other algorithms. In particular, our proposed method produced clustering results comparable to an algorithm that uses a complete dataset, and it outperformed an algorithm in which samples with missing data are removed and another algorithm in which missing values are imputed by mean replacement. The results are consistent with Hunt and Jorgensen’s reports [32]. Even with datasets in which the boundaries of the clusters overlap, such as datasets *A*_3_*B*_3_, *A*_2_*B*_4_, and *A*_3_*B*_4_, our algorithm performed well.

Furthermore, we found that the proposed algorithm outperformed the FCM clustering method on Fisher’s Iris dataset and on the Yeast Cell-cycle dataset. The FCM clustering method, a heuristic approach, has been verified as a successful missing-value clustering method [33]. FCM clusters data based on the characteristics of the samples. The criteria for FCM clustering are inconsistent, however, because the characteristics of dynamic algorithms lead to uncertainties about the samples, degrading FCM’s ability to precisely cluster data [34–36]. Our study shows that the proposed method is more likely to result in a low MR when the dataset is exactly a multivariable Gaussian mixture distribution. That is, it is more accurate than other methods with both simulated data and real data, e.g., Fisher’s Iris dataset and the Yeast Cell-cycle dataset. One possible reason for this is that our method estimates missing values with a conditional expectation based on the Gaussian mixture distribution and generates a “pseudo-complete” dataset. In addition, our method combines dynamic clustering with discriminant analysis and uses the EM algorithm to achieve a maximum-likelihood function. It can thus better utilize the information from known data to discover the intrinsic and implicit properties of a dataset. Usually, model-based methods are better than heuristic approaches, even though the former are always more time consuming. Model-based methods depend on a probability model, which is usually proposed based on the experience and feasibility of the model. The convergence and accuracy of the EM algorithm have already been established [25]. The Gaussian mixture model is both convenient and robust. Moreover, a model-based method provides substantial information—e.g., the posterior probabilities of individuals and the parameters of each cluster—and this information can be exploited to more accurately impute missing values with iterative updates to the algorithm until convergence to a minimizer is reach.

Our method uses the Bayesian posterior probability to cluster individuals; individuals are assigned to the cluster having the highest posterior probability. It is more reasonable to assign individuals to a cluster having a high posterior probability, such as 0.8 or 0.9, and this improves the clustering accuracy. Whereas traditional dynamic clustering methods cluster individuals directly to a certain category, this process is difficult when the boundaries of the clusters overlap. Another advantage to a model-based approach is that statistical criteria, such as AIC and BIC [14], can be used to discover the number of clusters that best suit the data. We confirmed the effectiveness of such criteria with results indicating three clusters for Fisher’s Iris dataset (a dataset with exactly three species of *Iris*) and five clusters for the Yeast Cell-cycle Gene-expression dataset. However, the reversible-jump Markov-chain Monte Carlo (MCMC) method was previously developed to infer the number of distributions in a mixture [37]. MCMC estimates the optimal number of clusters from a Bayesian perspective. Our future research shall compare MCMC with the BIC criteria used in this study. Finally, a prerequisite for raw data collected by experimenters is that they be normalized. Indeed, our method is more sensitive to data that have not been normalized. Therefore, either a log or some other form of data transformation should be applied in order to distribute the data in a normal fashion. Furthermore, because our proposal relies on the information from known individuals when imputing missing values, a large sample size is desirable for more accuracy when estimating missing values.

## Conclusions

In this paper, we have presented a model-based missing-data imputation algorithm for clustering numerical data with missing values. We achieved a high convergence rate with our algorithm when it was tested on simulated and real datasets. In addition, the statistical power of our algorithm is high, and it can accurately estimate parameters and correctly cluster individuals with missing variables. Moreover, the results of our evaluation show a low MR, even when the mean vectors of two clusters are close to each other or when their variance-covariance is disperse. The algorithm was applied successfully to datasets (both simulated and real) having missing data. Its performance is superior to that of an algorithm in which samples with missing data were removed and another algorithm in which missing values were imputed by mean replacement. A comparative evaluation demonstrated that our proposed algorithm outperforms the FCM method when the data from each cluster fit a multivariate Gaussian distribution.

## References

[1] Wylie MP, Holtizman J. The non-line of sight problem in mobile location estimation. In: Proc IEEE ICUPC. Cambridge. 1996; 2: 827–31. 10.1109/ICUPC.1996.562692

[2] Zhang YT, Fang KT. Introduction to Multivariate Statistical Analysis. Beijing: Science Press, 1983; 401–57.

[3] Johnoson RA, Wichern DW. Applied Multivariate Statistical Analysis. New Jersey: Prentice-Hall, Inc 1992; 532–560.

[4] Eisen M, Spellman P, Brown P, Botstein D. Cluster analysis and display of genome-wide expression patterns. Proceedings of the National Academy of Sciences. 1998; 95(25): 14863–8. 10.1073/pnas.95.25.14863 PMC24541 9843981

[5] Quackenbush J. Computational analysis of microarray data. Nature Reviews Genetics. 2001; 2(6): 418–27. 10.1038/35076576 11389458

[6] Speed T. Statistical Analysis of Gene Expression Microarray Data. London: Chapman & Hall/CRC Press 2003; 45–7.

[7] MacQueen J. Some methods for classification and analysis of multivariate observations. In: Proceedings of the 5th Berkeley Symposium. 1967; 1, 281–97.

[8] Hartigan JA, Wong MA. A K-means clustering algorithm. Journal of Applied Statistics. 1979; 28(1): 100–8. 10.2307/2346830

[9] Herrero J, Valencia A, Dopazo J. A hierarchical unsupervised growing neural network for clustering gene expression patterns. Bioinformatics. 2001; 17(2): 126–36. 10.1093/bioinformatics/17.2.126 11238068

[10] Selim SZ, Alsultan K. A simulated annealing algorithm for the clustering problem. Pattern Recognition. 1991; 24(91): 1003–8, 10.1016/0031-3203(91)90097-O

[11] Dasgupta A, Raftery AE. Detecting features in spatial point processes with clutter via model-based clustering. Journal of the American Statistical Association. 1998; 93: 294–302. 10.1080/01621459.1998.10474110

[12] McLachlan GJ, Basford KE. Mixture Models: Inference and Applications to Clustering. New York: Marcel Dekker, 1988.

[13] Titterington DM, Smith AFM, Makov UE. Statistical Analysis of Finite Mixture Distributions. New York: John Wiley & Sons, Inc, 1985.

[14] Schwarz G. Estimating the dimension of a model. Annals of Statistics. 1978; 6(2): 461–4. 10.1214/aos/1176344136

[15] Krzysztof S. Clustering with missing values. Fundamenta informaticae. 2013; 123(3): 331–50. 10.3233/FI-2013-814

[16] Rubin DB. Inference and missing data. Biometrika. 1976; 63(1): 581–92. 10.1093/biomet/63.3.581

[17] Rubin DB. Multiple imputations in sample surveys-a phenomenological Bayesian approach to nonresponse. Journal of the American Statistical Association. 1978; (1): 20–34. 10.1631/jzus.C10b0359

[18] Carpenter J, Kenward M. Multiple Imputation and its Application. New York: Wiley & Sons; 2012.

[19] Chen J, Shao J. Nearest neighbor imputation for survey data. Journal of Official Statistics. 2000; 16(2): 113–32.

[20] Yang J, Zhao Y, Ding WX. Missing data in survey sampling interpolation method. Applica Stat Manage (Chin). 2008; 27: 821–32.

[21] Hathaway RJ, Bezdek JC. Fuzzy c-Means Clustering of incomplete data. 2001; 31(5): 735–744. 10.1109/3477.956035 18244838

[22] Ghahramani ZB, Jordan MI. Supervised learning from incomplete data via an EM approach. Advances in Neural Information Processing Systems, 1994, 6: 120–127.

[23] Qu Y, Xu SZ. Supervised cluster analysis for microarray data based on multivariate Gaussian mixture. Bioinformatics. 2004; 20: 1905–13. 10.1093/bioinformatics/bth177 15044244

[24] Si YQ, Liu P, Li PH, Brutnell TP. Model-based clustering for RNA-seq data. Bioinformatics 2014; 30(2): 197–205. 10.1093/bioinformatics/btt632 24191069

[25] Hayes M, Pyon YS, Li J. A model-based clustering method for genomic structural variant prediction and genotyping using paired-end sequencing data. PLoS ONE. 2012; 7(12): e52881 10.1371/journal.pone.0052881 23300804 PMC3531386

[26] Wang SC, Li XL, Tang HY. Hybrid data clustering based on dependency structure and gibbs sampling. Lecture Notes in Computer Science 2006; 4304: 1145–51. 10.1007/11941439_138

[27] Dempster AP, Laird NM, Rubin DB. Maximum likelihood from incomplete data via the EM algorithm. Journal of the Royal Statistical Society Series B-statistical Methodology. 1977; 39(1): 1–38.

[28] Cho RJ, Campbell MJ, Winzeler EA, Steinmetz L, Conway A, Wodicka L, et al A genome-wide transcriptional analysis of the mitotic cell cycle. Mol. Cell. 1998; 2(1): 65–73. 10.1016/S1097-2765(00)80114-8 9702192

[29] Yeung KY, Fraley C, Murua A, Raftery AE, Ruzzo WL. Model-based clustering and data transformations for gene expression data. Bioinformatics. 2001; 17: 977–87. 10.1093/bioinformatics/17.10.977 11673243

[30] Krizhevsky A. Learning multiple layers of features from Tiny Images. Master’s thesis, Dept. of Comp. Sci., University of Toronto, 2009.

[31] Troyanskaya O, Cantor M, Sherlock G, Brown P, Hastie T, Tibshirani R, et al Missing value estimation methods for DNA microarrays. Bioinformatics. 2001; 17: 520–25. 10.1093/bioinformatics/17.6.520 11395428

[32] Hunt L, Jorgensen M. Mixture model clustering for mixed data with missing information. Computational Statistics & Data Analysis. 2003; 41(s3-4): 429–40. 10.1016/S0167-9473(02)00190-1

[33] Jiao YB, Liu HB, Zhang P, Wang XQ, Wei HB. Unsupervised performance evaluation strategy for bridge superstructure based on Fuzzy clustering and field data. The Scientific World Journal. 2013; 2013(2): 544–54. 10.1155/2013/427072 24288483 PMC3830814

[34] Sebzalli YM, Wang XZ. Knowledge discovery from process operational data using PCA and fuzzy clustering. Engineering Applications of Artificial Intelligence. 2001; 14(5): 607–16. 10.1016/S0952-1976(01)00032-X

[35] Podofillini L, Zio E, Mercurio D, Dang VN. Dynamic safety assessment: scenario identification via a possibilistic clustering approach. Reliability Engineering & System Safety. 2010; 95(5): 534–49. 10.1016/j.ress.2010.01.004

[36] Li SY. Engineering Fuzzy Mathematics with Application, Harbin: Harbin Institute of Technology Press; China; 2004.

[37] Green PJ. Reversible jump Markov chain Monte Carlo computation and Bayesian model determination. Biometrika. 1995; 363(4): 711–32. 10.1093/biomet/82.4.711

